# Fuzhuan Brick Tea Boosts Melanogenesis and Prevents Hair Graying through Reduction of Oxidative Stress via *NRF2*-*HO-1* Signaling

**DOI:** 10.3390/antiox11030599

**Published:** 2022-03-21

**Authors:** Peijun Zhao, Na Hyun Park, Md Badrul Alam, Sang-Han Lee

**Affiliations:** 1 Department of Food Science and Biotechnology, Graduate School, Kyungpook National University, Daegu 41566, Korea; zhaopeijun@henau.edu.cn (P.Z.); winner377@knu.ac.kr (N.H.P.); mbalam@knu.ac.kr (M.B.A.); 2 Department of Food Science and Technology, Henan Agricultural University, Zhengzhou 450046, China; 3 Food and Bio-Industry Research Institute, Kyungpook National University, Daegu 41566, Korea

**Keywords:** Fuzhuan brick tea, melanogenesis, hair graying, oxidative stress, NRF2, HO-1

## Abstract

The anti-graying effect of the hexane fraction of Fuzhuan brick tea is investigated in Melan-A cells and C57BL/6 mice. As a result, it is found that reactive oxygen species-induced damage is associated with the reduction of melanogenesis in hair bulb melanocytes when reactive oxygen species generation in Melan-A cells occurred. The results revealed that the hexane fraction of Fuzhuan brick tea could remarkably reduce reactive oxygen species generation in Melan-A cells; meanwhile, it could increase the cellular tyrosinase and melanin content, as well as up-regulate the expression of tyrosinase, tyrosinase related protein-1, tyrosinase related protein-2, and microphthalmia-associated transcription factor, and activate the MAP-kinase pathway through activating the phosphorylation of p38 c-Jun N terminal kinase/extracellular signal-regulated kinase. Furthermore, high-pressure liquid chromatography analysis reveals that the tea’s major ingredients in hexane fraction include gallic acid, theaflavin, theobromine, caffeine, epicatechin, and quercetin. Together, the current results suggest that Fuzhuan brick tea proves to protect from the damage of hydroquinone, which induces hair pigment loss.

## 1. Introduction

Hair color depends on the melanin content in the hair follicles, and a melanin deficiency in the follicles will cause the black hair to turn into gray hair. The pathogenic mechanisms of black hair turning into gray hair include melanin dysfunction and melanin loss, which may be related to genetics, pressure, environmental factors, nutritional status, and oxidative stress (OS) [1,2]. Typically, OS plays a pivotal role in increasing the damage to all tissues/biomolecules, including proteins and DNA. With the growth of hair, melanin is continuously synthesized in the follicle; during this process, tyrosine hydroxylation and _L_-3,4-dihydroxyphenylalanine (DOPA)-to-melanin oxidation will release high levels of reactive oxygen species (ROS) into the follicle. Wood et al. [3] revealed that H_2_O_2_ at millimolar concentrations was accumulated in the gray hair follicle of humans and the skin of vitiligo patients, causing the irreversible inactivation of tyrosinase since the methionine residues such as Met374 at its active site were oxidized. There are vacuolated melanocytes in the aging hair follicle, which is a typical OS-induced cellular response [4]. In the gray hair follicle, the presence of diverse stressors destroys the antioxidative system consisting of B-cell lymphoma-2 (Bcl-2), NF-E2-related factor2 (Nrf2), methionine sulfoxide reductase, catalase, and tyrosinase-related protein-2 (TRP2). As a result, the OS accelerates due to damage to the antioxidative system, causing selective premature senility and apoptosis of the hair follicle melanin stem cells [5,6]. In the black hair follicle, melanocytes with exuberant melanin secretion can express a high level of *Bcl-2*, which can survive in the endogenous OS induced by melanin production and ROS attack caused by ultraviolet radiation [6]. Nrf2 can be significantly expressed in the hair follicle’s epithelial tissues, including stromal keratinocytes, internal root sheath, hair follicle melanocytes, and outer root sheath [7]. Ko et al. [8] proposed that H_2_O_2_ reduced the expression of microphthalmia-associated transcription factor (MITF) in human melanocytes, but increased that of Nrf2 in cell nuclei, suggesting that H_2_O_2_ might play a key role in regulating the production and aging of MITF and Nrf2 in melanin production.

Fuzhuan brick tea (FBT) is a kind of brick tea; different from other kinds of brick tea, large amounts of *Eurotium Cristatum* (also called “golden flower”) will grow on the surface and in the interior of the brick tea after special fermentation, which is the predominant strain during the fermentation process of FBT. FBT’s varied biochemical profiles are thought to be important in producing the tea’s unique flavors and health benefits. Microbial fermentation boosted the anti-dysentery activity of FBT [9]. As a result, it is plausible to assume that microbial fermentation produces the taste and beneficial chemicals during the FBT manufacturing process. This is thought to be the most important aspect in the development of its sensory properties and health benefits [10]. Several components are created during the manufacturing of FBT, including catechin derivatives, flavonoids and their glycosides, phenolic acids, alkaloids, and terpenoids. Degradation, oxidation, condensation, structural alteration, methylation, and glycosylation are all linked to these reactions [11]. Multiple beneficial effects of FBT have been discovered, such as antioxidants [12], anti-obesity [13], gut microbial flora regulation [14], and improving disorders of glucose and lipid metabolism [15]. However, most studies only examined the water-soluble extract of FBT, while its fat-soluble components and physiological activities were rarely studied. However, the role of hexane extract of FBT (FBTH) in melanogenesis in hair has not been investigated.

Currently, domestic and foreign studies on melanin synthesis mainly focus on suppressing melanin production to develop whitening healthcare foods and cosmetics. In contrast, research on promoting melanin synthesis, especially hair-blackening-related research, is rare. Here, this paper investigated whether FBTH could affect melanogenesis and protect melanocytes from oxidative stress. This is of certain practical significance to the development of tea resources in China, and provides a scientific foundation for developing healthy foods and cosmetics for hair care.

## 2. Materials and Methods

### 2.1. Drugs and Chemicals

Arbutin, 3-isobutyl-1-methylxanthine (IBMX), L-DOPA (L-3,4-dihydroxyphenylalanine), sodium hydroxide, thiazolyl blue tetrazolium bromide (MTT), *O*-tetradecanoyl phorbol-13-acetate (TPA), 2,2’-azobis(2-amidinopropane) dihydrochloride (AAPH), 8-methoxypsoralen (8-MOP), 3-Isobutyl-1-methyl-2,6(1H,3H)-purinedione (IBMX), and hydroquinone were obtained from Sigma-Aldrich Co. (St. Louis, MO, USA). All other reagents and chemicals were high-grade and commercially available. Antibodies bought from Bioworld Technology (St. Louis Park, MN, USA) included anti-Tyrosinase, anti-TYRP-1, anti-TYRP-2, anti-*MITF*, anti-phospho-p38, and anti-p38, anti-phospho-ERK, and anti-ERK, anti-phospho-JNK, and anti-JNK.

### 2.2. Preparation of Tea Extract and HPLC Analysis

The FBT was bought from Anhua, Hunan Province in China. Voucher specimen (KNU2018-FBT) was stored at Food Enzyme Biotechnology Lab. After air-dying, 100 g tea powder was mixed with 15-folds of deionization water (D.W) for 24 h, and 10-folds of EtOH for six hours in a shaking incubator at 60 °C. Then, the supernatant was collected with a filter paper (No. 1 Whatman Schleicher & Schuell, Keene, NH, USA) and dried using a rotary vacuum evaporator (Tokyo Rikakikai Co. Ltd., Tokyo, Japan). The residue was considered an ethanolic extract, after which it was suspended in 200 mL of D.W, followed by mixing with n-hexane, chloroform, and ethyl acetate in sequence using separator funnels. After vacuum filtration, the samples were filtered and freeze-dried using a lyophilizer (MCTD85, Il-shin, Korea) and obtained n-hexane fraction (FBTH), chloroform fraction (FBTC), and ethyl acetate fraction (FBTE). Finally, the fractions and aqueous extract (FBTA) were lyophilized and dissolved in dimethyl sulfoxide (DMSO) or D.W at concentrations of 1, 3, 10, 30, or 100 mg/mL. The phytochemical features of FBTA, FBTE, FBTC, FBTH and the standard compounds gallic acid, theaflavins, theobromine, epigallocatechin, caffeine, epicatechin, epigallocatechin gallate and quercetin were identified using a High-Performance Liquid Chromatograph (HPLC) with a Shimadzu Prominence AutoSampler (SIL-20A) HPLC system (Shimadzu, Kyoto, Japan). Reverse-phase chromatographic analysis was conducted using a Phenomenex C18 column (4.6 mm × 250 mm) packed with 5-μm diameter particles. A stepwise gradient of solvent A (1% formic acid solution) to solvent B (acetonitrile) was used with the ratio changing each minute as follows: 10% A up to ten minutes, which was then changed to obtain 30%, 50%, 60%, 90%, 20%, and 10% A in 15, 20, 25, 30, 35, and 40 min, respectively, at λ = 280 nm. Polyphenolic compounds were identified by comparing retention times with those of available pure standards.

### 2.3. Cell Culture and Cell Viability Assay

Melan-A cells were obtained from Dorothy C. Bennett (St George’s, University of London, London, UK), and cultured using offered method [16]. The cells were cultured in RPMI 1640 medium (Cellgro, Lincoln, NE, USA) containing 10% fetal bovine serum (FBS), streptomycin-penicillin (100 µg/mL each), and 200 nM TPA (a potent tumor promoter) at 37 °C in 5% CO_2_, and the cells were sub-cultured every three days. Additionally, the cell viability test was performed using the MTT method to confirm the cytotoxicity of FBTH.

### 2.4. Cellular Melanin Contents in Melan-A Cells

Melan-A cells (1 × 10^5^ cells/mL) were seeded into a 24-well plate (BD Falcon, Bedford, MA, USA) and cultured overnight. Then, the FBTH (3, 10, 30 μg/mL) was added and incubated for three days. The medium was changed to a fresh medium containing FBTH every day. After washing with cold phosphate buffer saline phosphate-buffered saline (PBS), the cells were dissolved in 1 N NaOH, and the absorbance was read at 405 nm using a spectrophotometer (VICTOR3, Perkin Elmer, Waltham, MA, USA). IBMX was used as a positive control, while arbutin was used as a negative control.

### 2.5. Zymography Analysis of Intracellular Tyrosinase

Tyrosinase zymography was executed as described previously [17]. Melan-A cells (1 × 10^5^ cells/mL) were cultured with FBTH, arbutin, and IBMX in 6-well plates for three days. Then, the cells were washed with PBS and collected with radioimmunoprecipitation assay (RIPA) buffer. The protein was extracted and unified using bicinchoninic acid (BCA) method, and 40 μg protein from every sample separated using 10% sodium dodecyl sulfate-polyacrylamide gel electrophoresis (SDS-PAGE) according to its molecular weight. Then, the gel, which contained the enzymes was incubated in 0.1 M sodium phosphate buffer pH 6.5 with 5 mM L-DOPA in the dark at 37 °C for one hour. Finally, the macroscopic bands in the gel were quantified using Image Lab™ Software, version 5.2.1 (Bio-Rad Laboratories, Hercules, CA, USA).

### 2.6. Reverse-Transcription Polymerase Chain Reaction (RT-PCR) Analysis

Total RNA was extracted from homogenized cells and mouse skin tissue using TRIzol kit (Invitrogen, Carlsbad, CA, USA). Two µg total RNA was used to prepare the complementary DNA (cDNA) using RT & GO MasterMix (MP Biomedicals, Aurora, OH, USA). The other steps were performed as the methods described [16]. The primer sets used in this study were as follows: Tyrosinase, forward (F): 5′-CCC AGA AGC CAA TGC ACC TA-3′ and reverse (R): 5′-ATA ACA GCC CCA CCA GTG C-3′; *TRP-1*, F: 5′-GCT GCA GGA GCC TTC TTT CT-3′ and R: 5′-AGA CGC TGC ACT GCT GGT CC-3′; *TRP-2*, F: 5′-GGATGACCGTGAGCAATGGC-3′ and R: 5′-CGG TTG TGA CCA ATG GGT GC-3′; *MITF*, F: 5′-CAG GCT AGA GCG CAT GGA CT-3′ and R: 5′-CTC CGT TTC TTC TGC GCT CA-3′; *GAPDH*, F: 5′-TTG TGA TGG GTG TGA ACC AC-3′, and R: 5′-ACA CAT TGG GGG TAG GAA CA-3′. The products were electrophoresis in 1% agarose gels at 100 V for 25 min and stained by EtBr (Bio-Rad Laboratories, Hercules, CA, USA) staining. The bands were quantified using Image Lab™ Software, version 5.2.1 (Bio-Rad Laboratories, CA, USA).

### 2.7. Western Blot Analysis

Melan-A cell or mouse skin tissue lysates were mixed with RIPA buffer and denatured at 100 °C for five minutes using a standard protocol. 10% of SDS-PAGE was used to separate the sample proteins (50 µg). Following electrotransfer to nitrocellulose membranes (Whatman, Dassel, Germany), the membranes were immersed overnight in a mixture containing primary antibodies, and 5% skim milk. After washing with tris buffered saline with 0.1% Tween 20 detergent (TBST), specific secondary antibody was added and finally the resulting reaction was exposed using an ECL solution system (Perkin Elmer). The protein levels were quantified using the Image Lab™ software, version 5.2.1 (Bio-Rad Laboratories, Hercules, CA, USA).

### 2.8. Animals and Care

Seven-week-old male C57BL/6 mice were obtained from Central Laboratory Animals, Inc. (Seoul, Korea) and housed in an air-conditioned animal room at a temperature of 23 ± 1 °C, a humidity of 55 ± 5%, and 12/12 h light/dark cycle with *ad libitum* access to water and standard laboratory diet. The animals were acclimatized for one week and were randomly divided into five groups consisting of five mice per group. The experiment was performed according to the guidelines for animal experiments issued by Kyungpook National University and approved by the Institutional Animal Care and Use Committee of Kyungpook National University (KNU-2018-0052).

### 2.9. Hydroquinone-Induced Grey Hair Model

**Figure 1:**
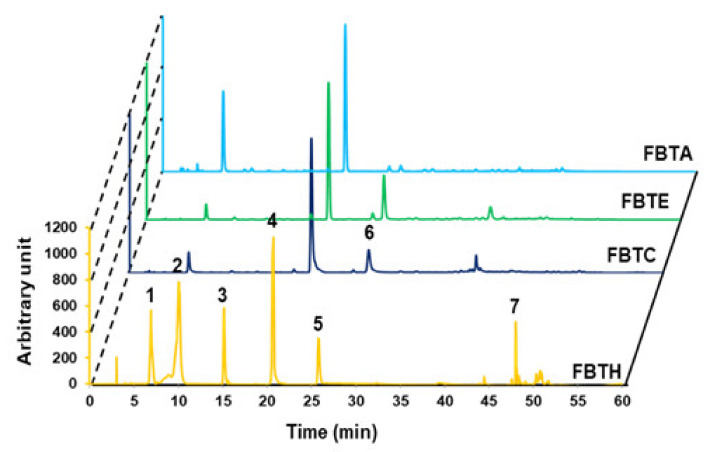
Analytical analysis by HPLC. HPLC analysis was performed to confirm the food ingredient in FBTH. The detailed conditions for high-performance liquid chromatography are explained in the Materials and Methods section. HPLC (absorbance at 280-nm)—profile of phenolic standards and FBTH. Peaks: gallic acid (1); theaflavin (2); theobromine (3); caffeine (4); epicatechin (5); epi-gallocatechin gallate (EGCG) (6); quercetin (7). The injection volume was 20-µL, and the flow rate was maintained at 0.8-mL/min. All tests were performed in triplicate.

The mice were randomly assigned to 5 groups (*n* = 5). As indicated in Table 1, Group A was the non-treated (NT) group with a solution of propylene glycol and ethanol (3:7) as a vehicle. After topically applying 8-MOP (positive control, 4.5 mg/kg/day), FBTH-L (FBTH low concentration, 50 mg/kg/day) or FBTH-H (FBTH high concentration, 100 mg/kg/day) for 30 min, all animals were applied with hydroquinone (HQ) except group A. All reagents were given through topical applications. This treatment was continued for two months until there was a visible difference between group A and group B. After 24 h of the last treatment, all animals were sacrificed, and the skin and hair were harvested for further experiments (Figure 1).

### 2.10. Melanin Content Analysis in Hair Shaft

Hair samples of all mice were collected on the last day of the experiment. All collected hair samples were washed with deionized water twice to remove sample color and impurities. After drying, the hair samples of every mouse were weighted consistently, then dissolved in 1 N NaOH, and heated to 80 °C overnight until the hair dissolved completely. Then, the absorbance of the supernatant at 405-nm was measured thrice using a UV-spectrophotometer (Victor3, PerkinElmer, Waltham, MA, USA).

### 2.11. Fontana–Masson Staining

Fontana-Masson staining method described previously [16] was conducted to assess the hair follicle melanin formation in the skin of C57BL/6 mice. Fresh skin samples were fixed in 4% paraformaldehyde overnight at 27 ± 1 °C and stained using a Fontana-Masson staining kit (American MasterTech, Inc., Lodi, CA, USA). Sliced skin samples were briefly stained with an ammoniacal silver solution for 60 min at 60 °C. The samples were incubated in 0.1% gold chloride, followed by incubation in 5% sodium thiosulfate. Melanin spots were visualized using an AE-31 light microscope (Motic Asia, Kowloon, Hong Kong, China).

### 2.12. Image Analyses and Quantification

Microscope photos of Melan-A cells, hair-graying area of mice dorsum, and hair shafts were analyzed using ImageJ, Version 1.8.0. Briefly, brightness and staining intensities were measured for equal areas as mean pixel intensity. Results were normalized and expressed as fold change compared to controls.

### 2.13. Statistical Analysis

The data were analyzed using GraphPad Prism software (version 5, San Di ego, CA, USA) using one-way ANOVA, followed by the Tukey post hoc test after the normality and homogeneity of the variance test were checked. A value of (*p* < 0.05, *p* < 0.01, *p* < 0.001) was significant for the differences.

## 3. Results

### 3.1. HPLC Analysis of FBTH

The major polyphenolics in FBTH were detected using high-performance liquid chromatography (HPLC) with various standards. As described in Figure 1, the chromatogram shows that FBTH exhibited peaks with the same retention times as the following standard polyphenolics: gallic acid (6880 min), theaflavin (10,053 min), theobromine (15,153 min), caffeine (20,580 min), epicatechin (25,809 min), and quercetin (47,999 min). The number of these polyphenolic compounds in HPLC was analyzed by applying the peak areas of the standards with known concentrations.

### 3.2. Attenuation of Cellular Oxidative Stress by FBTH

**Figure 2:**
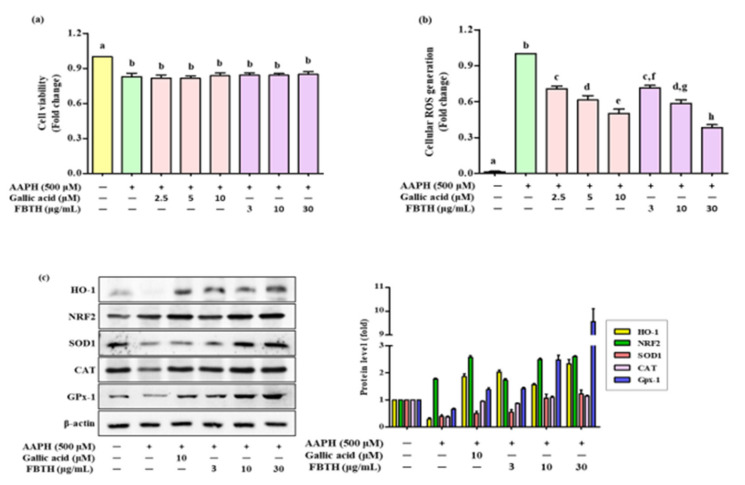
Effects of cellular ROS generation and antioxidant enzyme expression induced by AAPH in FBTH-treated Melan-A cells. (**a**) Cells were incubated using FBTH overnight and treated with AAPH, followed by another 24 h incubation. Cell viability was measured by MTT analysis. (**b**) Cellular ROS were measured according to the 2’,7’-dichlorofluorescin (DCF) fluorescence intensity in Melan-A cells. (**c**) Analysis of primary and phase II antioxidant and detoxifying enzymes. Melan-A cells were pretreated with 2-µL of FBTH for 24 h. Protein expressions of phase I and phase II antioxidant enzymes analyzed by Western blotting and compared to the band intensity were also shown in the adjacent figure. Data are expressed as the mean ± SD (*n* = 3). All tests were performed in triplicate. Different letters stand for statistically significant each other (*p* < 0.05) performed by one-way ANOVA followed by Tukey’s test. HO-1, heme oxygenase-1; NRF2, NF-E2-related factor2; SOD-1, superoxide dismutase-1; CAT, catalase; GPx-1, glutathione peroxidase-1.

To determine the protecting effect of FBTH in Melan-A cells from oxidative stress, 2,2’-Azobis (2-amidinopropane) dihydrochloride (AAPH) was used to contribute the oxidative stress in an in vitro model. AAPH is an extensively reported water-soluble azo-free radical generator. It is considered a pro-oxidant model molecule and is widely used to assess the mechanisms of oxidative stress-induced cellular imbalance in cells or tissues [18]. Melan-A cells treated with FBTH did not induce any cellular toxicity after the APPH treatment and maintained cell viability of more than 80% (Figure 2a), while ROS formation was reduced by 28.6%, 41.8%, and 61.2% with different concentrations (3, 10, and 30-µg/mL) of FBTH, respectively (Figure 2b). These results suggest that FBTH has a strong effect on antioxidant potential.

### 3.3. Effects of FBTH on Antioxidant Enzyme Expression in Melan-A Cells

To investigate the effects of FBTH on antioxidants (SOD1, CAT, and GPx-1) and phase II detoxifying enzymes HO-1 the cells were treated using FBTH at 3, 10, and 30 μg/mL for 24 h. Western blotting indicated a dose-dependent increase in the protein levels of SOD1, CAT, and GPx-1 (Figure 2c) and phase II detoxifying enzymes HO-1 and its transcription factor Nrf2. The band intensity of each antioxidant enzyme expression was compared. Results indicated that the expression of HO-1 and GPx-1 was dramatically increased by 2.8- and 9.5-fold, respectively (Figure 2c adjacent figure). These data indicate that the antioxidant activity of FBTH might be related to activating primary antioxidant enzymes and phase II detoxifying enzymes by increasing their expression at the protein level.

### 3.4. Effects of the FBTH on Melanogenesis in Melan-A Cells

**Figure 3:**
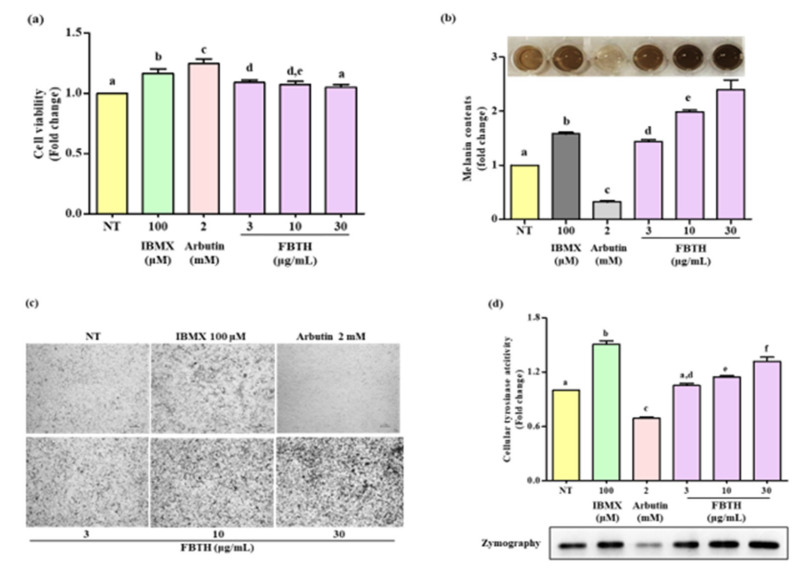
Effects of melanogenesis by FBTH in Melan-A cells. (**a**) Cells (1 × 105 cells/mL) were seeded in 24-well plates and incubated with the FBTH or IBMX or arbutin for three days, and the cell viability was measured by the MTT assay. (**b**) Cells were treated with samples or compounds for three days; after washing with PBS twice, the cells were collected and dissolved in NaOH, then the melanin contents were measured at 405 nm. IBMX was used as a positive control, and arbutin was used as a negative control. (**c**) Cells were treated with samples or compounds for three days, and then washed using PBS twice and photographed using a Nikon microscope and camera (Eclipse, TE-2000U, Nikon, Tokyo, Japan). (**d**) Melan-A cells were cultured with FBTH for three days. After the tyrosinase was extracted, the cellular tyrosinase activity and zymography were performed according to the described method. Data are expressed as the mean ± SD (*n* = 3). All tests were performed in triplicate. Different letters stand for statistically significant each other (*p* < 0.05) performed by one-way ANOVA followed by Tukey’s test.

The Melan-A cell line was a normal counterpart to melanoma cells and was steadily used for evaluating the melanogenesis effect. It was found the non-toxic dose of FBTH (Figure 3a) significantly increased melanin production in a dose-dependent manner (Figure 3b). The microscopic features of the cells are indicated in Figure 3c, indicating that the melanin content in the cells was increased by FBTH treatment. As shown in Figure 3d, FBTH treatment significantly induced cellular tyrosinase activity in a dose-dependent manner, which was supported by L-DOPA zymography analysis. These findings suggest that melanin content was accelerated by FBTH treatment in Melan-A cells.

### 3.5. Effect of FBTH on the Expression of Melanogenesis-Related Proteins

**Figure 4:**
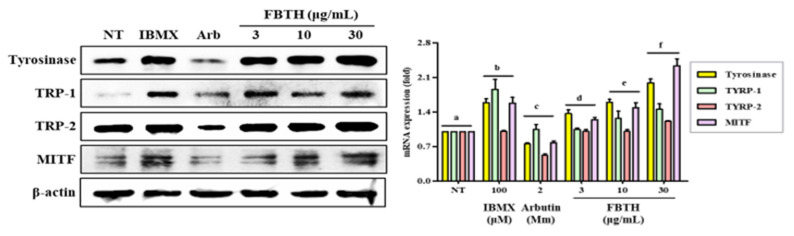
Effects of the FBTH on the expression of melanogenesis-related proteins in Melan-A cells. Cells (1 × 105 cells/mL) were cultured with FBTH for 72 h, and the protein levels were analyzed by Western blotting, and the statistical analysis of protein band density was shown. IBMX (100 μM) was used as the positive control, and arbutin (2 mM) was used as the negative control. NT: No treatment. Data are expressed as the mean ± SD (*n* = 3). All tests were performed in triplicate. Different letters stand for statistically significant each other (*p* < 0.05) performed by one-way ANOVA followed by Tukey’s test.

To further investigate the mechanism of promoting melanogenesis, protein levels of tyrosinase, TRP-1, TRP-2, and MITF were measured by Western blotting. Compared with untreated control cells, FBTH treatment at 30-μg/mL significantly augmented up to 2.0-, 1.5-, 1.3-, and 2.65-fold increase in the tyrosinase, TYRP1, TYRP-2, and MITF protein levels, respectively (Figure 4, first–fourth lane; compare between first lane and third lane or fourth–sixth lane).

### 3.6. Effects of FBTH on the Melanogenesis-Associated Signaling Pathways

**Figure 5:**
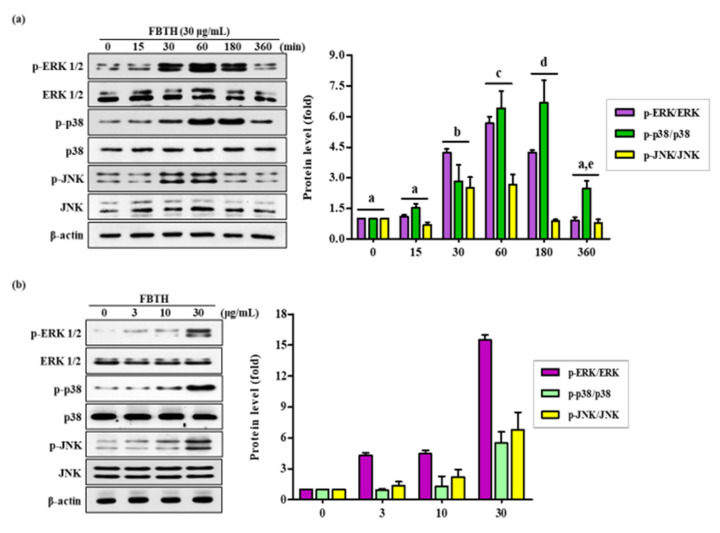
Effects of the FBTH on the expression of ERK/p38/JNK phosphorylation in Melan-A cells. Cells (1 × 105 cells/mL) were cultured with FBTH (3, 10, and 30-μg/mL) for 72 h, and the protein levels were analyzed by Western blotting (**a**,**b**). The statistical analysis of protein band density was shown in the adjacent figure in a time (**a**) or concentration (**b**)-dependent manner. NT: No treatment. Data are expressed as the mean ± SD (*n* = 3). All tests were performed in triplicate. Different letters stand for statistically significant each other (*p* < 0.05) performed by one-way ANOVA followed by Tukey’s test.

Melanogenesis is controlled by various signaling pathways. Many pieces of evidence suggest that phosphorylation of p38/JNK mitogen-activated protein kinase (MAPK) can increase melanogenesis by activating MITF. Thus, to investigate the mechanism of FBTH-induced melanogenesis, MAPK phosphorylation was estimated in Melan-A cells after 24 h treatment with FBTH at the indicated concentrations (3–30 μg/mL). As expected, p38 phosphorylation was stimulated by FBTH in a concentration-dependent manner from 60 min to three hours after treatment (Figure 5a). These findings suggest that the MAPK signaling pathway is involved in the effect of FBTH-induced melanogenesis.

### 3.7. Topical Application of FBTH Improves Hydroquinone-Induced Hair Graying in C57BL/6 Mice

**Figure 6:**
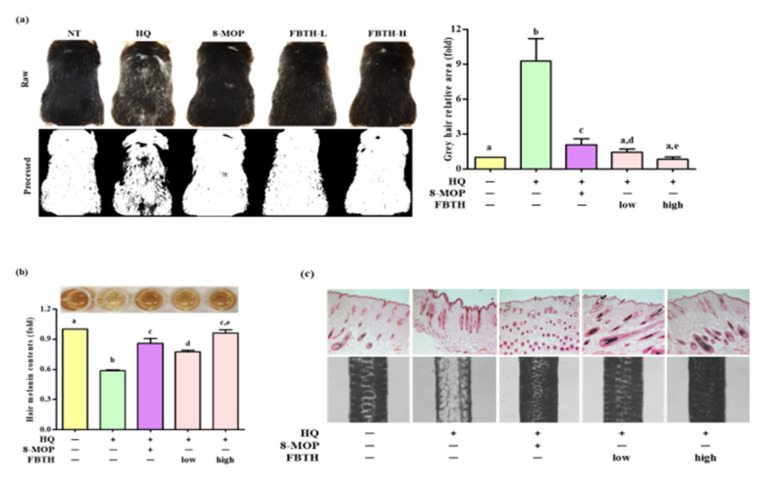
Repression of hair graying on C57BL/6 mice by FBTH. (**a**) After exposure to FBTH with or without HQ for two months, the whole mice were photographed using a Nikon camera (Raw). The gray hair coverage of the whole mouse dorsum was processed as a binary image using ImageJ software (processed). 8-MOP was used as a positive control. NT, vehicle group; HQ, hydroquinone control group; FBTH-L, low concentration (50 mg/kg/day) of the FBTH group; FBTH-H, high concentration (100 mg/kg/day) of the FBTH group. ImageJ software was used to quantify the gray hair area. (**b**) Melanin contents of C57BL/6 mice hair shafts. At the end of the schedule, hair shafts were collected and washed twice using deionized water to remove the sample color and impurities. The hair was dissolved in NaOH, and melanin content was measured at 405 nm after clear drying. (**c**) Fontana-Masson staining of dorsal skin sections from the mice showed differences in hair follicle melanin content (top). Melanin biosynthesis in the hair shafts of C57BL/6 mice (bottom). Three independent data are collected, and the classical feature is shown. For statistical analysis, different letter indicated that they are statistically significant each other.

The hair graying effects of FBTH in vivo were examined in C57BL/6 mice, which were treated according to the schedule indicated in Table 1. It was found that repeated hydroquinone exposure led to visible hair graying in the mice (Figure 6a, second photos of first and second lanes). As shown in Figure 6a, dramatic decolorization of mice’s hair was observed in the control group after hydroquinone treatment, but limited white-gray hair was discerned in the untreated group and FBTH-treated mice. ImageJ software was used to quantify the gray hair area of the mouse dorsum (Figure 6a, second lanes). After fading with hydroquinone, the total gray hair area was significantly increased in the model control group (Figure 6a, adjacent figure). However, this fading could be reversed by treatment with FBTH-L and FBTH-H. Histochemical analysis confirmed the effects of FBTH (Figure 6c).

**Figure 7:**
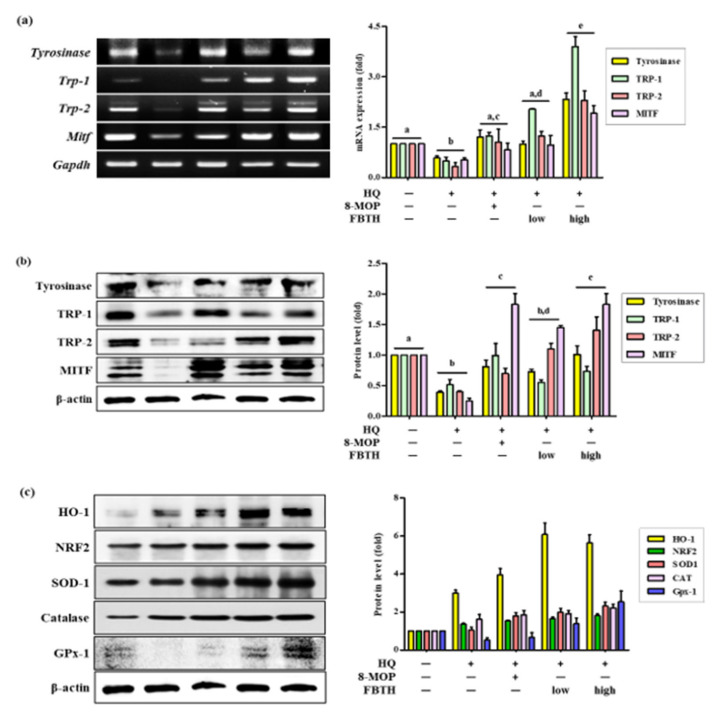
Comparison of mRNA and protein expression of melanogenesis and antioxidant related genes on hydroquinone-induced hair graying in C57BL/6 mice. (**a**–**c**) C57BL/6 mice were treated with FBTH and hydroquinone every day for two months. Then, the dorsal skin was collected, and the mRNA (**a**) or protein expressions (**b**) of melanogenesis related gene and protein expression of antioxidant related genes (**c**) were analyzed by RT-PCR and immunoblotting. Respectively. 8-MOP was used as a positive control. NT, vehicle group; HQ, hydroquinone control group; FBTH-L, low concentration (50 mg/kg/day) of the FBTH group; FBTH-H, high concentration (100 mg/kg/day) of the FBTH group. For statistical analysis, different letter indicated that they are statistically significant each other.

Hydroquinone-treated hair follicles and hair shafts exhibited decreased melanin staining (Figure 6c). Consistent with the animal dorsal grey hair area data, the FBTH-treated animals exhibited enhanced melanin staining compared to that of the hydroquinone-treated controls. Moreover, the hair shafts of C57BL/6 mice were obtained, observed by a Nikon microscope and camera (Eclipse, TE-2000U), and recorded with photos. Additionally, the hair shafts were dissolved in 1 N NaOH, and the absorbance was read at 405 nm by a microplate reader. The results showed that the total melanin content of the FBTH-L and FBTH-H groups was 32.9% and 46.4% higher than the model control group, respectively (Figure 6b). Additionally, hydroquinone treatment substantially decreased the expression of melanogenesis-related mRNA (Figure 7a) and proteins, including TYR, TRP-1, TRP-2, and MITF, compared to that of the control group (Figure 7b). These data suggest that FBTH can improve the hydroquinone-induced hair depigmentation in mice, apparently through increased melanogenesis.

### 3.8. Effects of FBTH on Antioxidant Enzyme Expression in Mice

The antioxidant enzyme expression in mouse skin tissue was also investigated. As shown by Western blotting, FBTH treatment upregulates the expressions of HO-1, NRF2, SOD-1, Catalase, and GPx-1 (Figure 7c and adjacent bar diagram). These results indicate that FBTH efficiently activates antioxidant enzyme activity in mouse skin.

## 4. Discussion

In this study, the regulatory effects of FBTH on Melan-A cells and C57BL/6 mice were investigated. It was found that FBTH could enhance melanogenesis in Melan-A cells and promote hair pigmentation in C57BL/6 mice. Subsequent studies showed that FBTH upregulated tyrosinase, TYRP-1, TYRP-2, and MITF expressions in cells- and mice-basis and activated the MAPKs-signaling pathway. The results also showed that FBTH could attenuate the oxidative stress in Melan-A cells and C57BL/6 mice induced by AAPH or hydroquinone. They alleviated the ROS scavenging by activating the NRF2/HO-1 antioxidant pathway.

Hair melanins are synthesized by hair melanocytes, located in the bulb of the hair follicle, which transfer pigments to the hair shaft, thus defining the hair pigmentation. The mechanisms of depigmentation in the hair follicle are currently unclear. Still, it has been reported that ROS overload plays a crucial role in depigmentation, which is associated with the imbalance between the pro- and antioxidant systems [19]. Now, the NRF2/ARE pathway, which includes antioxidative enzymes such as HO-1, SOD, catalase, and GPx1, impairment, is involved in this imbalance [20]. Increasing evidence reveals that the accumulation of H_2_O_2_, the absence of catalase and methionine sulfoxide reductase (MSR) protein in human white scalp hair shafts is associated with gray hair follicles [3]. Furthermore, tyrosinase and methionine sulfoxide residues melanocyte stem cells located in the hair follicle were considered targets for oxidation. Therefore, first the cellular antioxidant ability of FBTH was investigated. Interestingly, FBTH suppressed the ROS generation induced by AAPH in a dose-dependent manner in Melan-A cells (Figure 2b). Then, the protein levels of antioxidant enzymes were also investigated in cells and C57BL/6 mice. Hydroquinone is a major oxidant in tobacco smoke and atmospheric pollutants, generating ROS and promoting oxidative stress [21]. It was found that FBTH suppressed the oxidative stress induced by both AAPH and hydroquinone through augmentation of the expression of SOD, catalase, GPx-1, and HO-1 (Figure 2c and Figure 7c). Jiang et al. found that berberine (BBR) pretreatment induced Nrf2 nuclear translocation to increase the Nrf2 level and increase ARE activity, thus significantly decreasing the H_2_O_2_-induced ROS accumulation in human melanocytes and cell apoptosis; besides, it regulates MITF and its target protein to promote melanin production [22]. Sextius et al. suggested that *Polygonum multiflorum* extract (PME) reduced the H_2_O_2_-induced ROS accumulation in melanocytes and significantly enhanced the melanin level in human hair follicles in vitro [23]. Rojo et al. [24] used C57BL/6J (*Nrf2*^+/+^ vs. *Nrf2*^-/-^) transgenic mice to construct the ultraviolet-induced in vitro gray hair model and discovered that bixin activated Nrf2 to protect the ultraviolet-induced light injury and PUVA (psoralen + UVA)-induced hair pigment loss. Taguchi et al. [25] discovered that the North American *Eriodictyon* extract (Ea) rich in flavonoids activated the WNT/MITF/tyrosinase signaling pathway to significantly increase melanin synthesis in melanoma cells; moreover, human experiments also verified that Ea reduced gray beard and gray hair numbers in the subjects. Likewise, SOD prevented the damage to melanocyte DNA by decomposing superoxide, thereby suppressing the hair graying induced by PUVA in New Zealand Black mice [26].

Difference in skin pigmentation depends on oxidized/reduced hemoglobin (red/blue), carotenoids (yellow), and melanin (brown). Hair color relies only on the presence or absence of melanin [4]. Skin and hair follicle melanin are formed in melanosomes produced by melanocytes, and they share melanogenesis mechanisms in the same way, which are mediated by MITF-regulated tyrosinase and related proteins (TRP-1 and TRP-2). Moreover, the age-induced lack of pigment production is associated with the decline of the number of bulbar melanocytes in hair follicles, the presence of melanocytes within the outer root sheath of white hairs, and low DOPA-oxidase activity [27]. Tyrosinase is the first enzyme necessary in melanin biosynthesis and is responsible for the pigmentation of skin and hair in mammals. Some studies have revealed that tyrosinase mutation causes reduced melanin production and graying in rabbits [28] and made albino phenotype in laboratory mice [29] but reversed by introducing a functional tyrosinase minigene [30]. The expression of tyrosinase, TRP-1, and DOPA-oxidase activity was detected in the hair bulb melanocytes during the anagen phase of the hair growth cycle, and early loss of hair bulb melanocytes has been observed in mice with a mutation in TRP-1 [31]. It is well documented that the mutation or experimental deletion of TRP-2 in mice results in a diluted coat color and reduced melanin content in the hair shafts [32]. TRP-2 is also involved in cellular responses to oxidative stress. TRP-2 overexpression in melanoma cells increased GSH levels and reduced ROS-induced DNA damage, indicating that TRP-2 are capable to mitigate oxidative stress [33]. Recent studies have demonstrated that MITF mutations in mice cause the differentiation of premature melanocyte stem cells, resulting in hair graying [34]. Thus, the investigation of the mechanisms of the FBTH-induced anti-hair graying effect in vitro and in vivo was attempted. The melanogenesis-related experiments indicated that FBTH dose-dependently promoted the melanin content and tyrosinase activity in Melan-A cells (Figure 4). Hydroquinone (HQ) is one of the most effective melanogenesis inhibitors and have an excellent inhibitory ability of tyrosinase in vitro and in vivo. The agent is widely used for treating hyperpigmentation-related diseases [35]. Topical hydroquinone can induce obvious hair depigmentation in C57BL/6 mice [36]. In this study, hydroquinone was used as a hair graying inducer. As expected, dramatic decolorization of mice hair was observed in the control group after hydroquinone treatment (Figure 6a, second photo of raw and processed lanes). However, FBTH resisted the hair graying induced by hydroquinone (Figure 6a), and both low and high dose treatments of FBTH significantly augmented the tyrosinase, TRP1, TRP-2, and MITF levels in the mouse model (Figure 7a,b). It was reported that various natural or synthetic compounds, such as apigenin and luteolin, could promote melanogenesis [37]. However, theaflavin, theobromine, and caffeine at their putative concentrations in Fuzhuan brick tea increased the melanin content in Melan-A cells (Supplementary Material Figure S1). Our report is supported by Yamaoka et al. [38], who stated that theaflavin increased melanin in B16 melanoma cells. Furthermore, quercetin, one of the active compounds of FBT, enhances melanogenesis by increasing tyrosinase activity in human melanoma cells and normal human melanocytes [39,40]. Therefore, it can be assumed that caffeine, theaflavin, theobromine, and quercetin were collectively enhancing melanin synthesis in mouse hair follicles. In addition, Park et al. reported that *Pueraria thunbergiana* and its active compound, puerarin, significantly increased the melanin content in the hair shafts of gray-haired MITF*^vit/vit^* mice by enhancing the expression of tyrosinase and TRP-2 through up-regulation of MITF transcription [41]. 

Melanogenesis is controlled by various signaling pathways. Many pieces of evidence suggest that the phosphorylation of MAPKs can increase melanogenesis by activating MITF. In this study, FBTH activated the MAPKs (ERK, p38, and JNK) (Figure 5a,b). Increasing evidence has shown that phosphorylation of p38 responds to UVB-induced expression of MC1R and MITF in the pigmentation cascade, sequentially mediating UVB-induced tyrosinase expression [42,43,44,45]. Some studies have reported that melanogenesis enhancers increase the expression of tyrosinase, TRP-1, and TRP-2 by regulating p38 MAPK and MITF [16,17,44,45]. Thus, the effects of FBTH on boosting the melanogenesis process may be associated with the activation of MAPKs, resulting in the activation of MITF and melanogenesis-related proteins such as tyrosinase, TRP-1, and TRP-2.

## 5. Conclusions

Taken together, the current data strongly suggest that FBTH can significantly reduce oxidative insulted ROS generation by increasing the expression of antioxidant enzymes as well as stimulate melanogenesis by increasing the expression of tyrosinase, TRP-1, TRP-2, and MITF via MAPK signaling activation. Thus, it is suggested that FBTH is deserving of development as a food element capable of reversing hair pigment loss. Further research on the anti-graying potential of FBTH, not FBTA, should be directed toward developing food biomaterials for the use of inner beauty or cosmeceuticals, with a focus on the exploration of its bioactive molecules operating synergistically for anti-graying capabilities.

## Figures and Tables

**Table 1:** Animal grouping and treatments.

Groups	Reagent	Treatments
A (NT)	Vehicle	/
B (Control)	HQ, 200 mg/kg/day	/
C (Positive control)	HQ, 200 mg/kg/day	8-MOP, 4.5 mg/kg/day
D (Sample low)	HQ, 200 mg/kg/day	FBTH-L, 50 mg/kg/day
E (Sample high)	HQ, 200 mg/kg/day	FBTH-H, 100 mg/kg/day

HQ: Hydroquinone.

## Data Availability

The data presented in this study are openly available.

## Supplementary Materials

The following supporting information can be downloaded at: https://www.mdpi.com/article/10.3390/antiox11030599/s1, 1, Figure S1: Effect of caffeine, theaflavin and theobromine on melanin production in Melan-a cells.
