# Supplementary material for: Fuzhuan Brick Tea Boosts Melanogenesis and Prevents Hair Graying through Reduction of Oxidative Stress via NRF2-HO-1 Signaling
Source: Antioxidants (Basel). 2022 Mar 21;11(3):599. doi: 10.3390/antiox11030599 (PMC8945210; doi:10.3390/antiox11030599)
Supplement: Supplementary file 1 [file antioxidants-11-00599-s001.zip › antioxidants-1636325-supplementary.pdf]

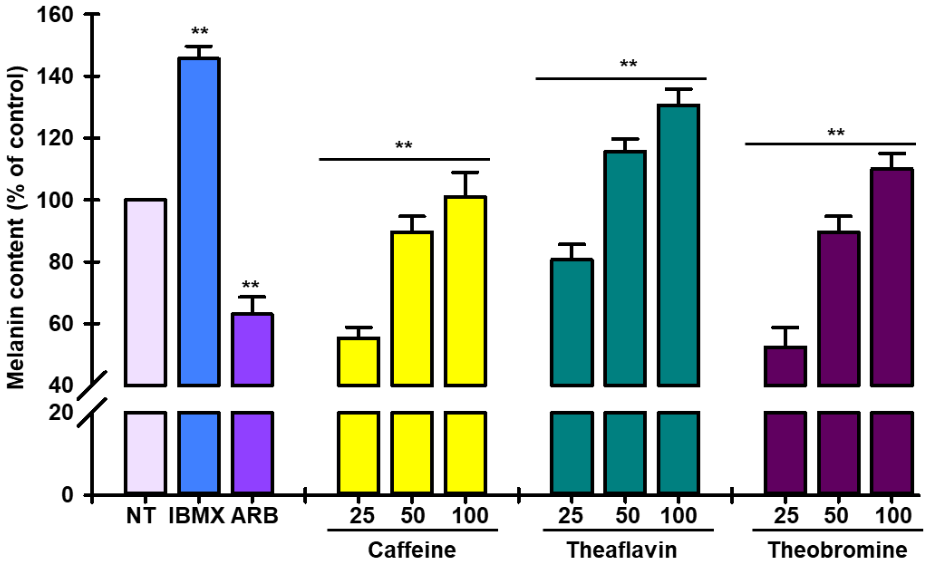

Figure S1: Effect of caffeine, theaflavin and theobromine on melanin production in Melan-a cells. Melan-a Cells ($1×10^5$ cells/mL) were seeded into a 24-well plate and cultured overnight. Then, the IBMX, arbutin, caffeine, theaflavin, or theobromine was added and incubated for three days. The medium was changed to a fresh medium containing each compound every day. After washing with cold PBS, the cells were dissolved in 1 N NaOH, and the absorbance was measured at 405 nm using a spectrophotometer (Victor3, PerkinElmer, Waltham, MA, USA). IBMX was used as a positive con trol, while arbutin was used as a negative control. ARB: arbutin. Data are expressed as the mean ± SD (n = 3). All tests were performed in triplicate. ** $p < 0.05$ performed by one-way ANOVA followed by Tukey 's test.
